# Delayed Enucleation With Neoadjuvant Chemotherapy in Advanced Intraocular Unilateral Retinoblastoma: AHOPCA II, a Prospective, Multi-Institutional Protocol in Central America

**DOI:** 10.1200/JCO.18.00141

**Published:** 2019-09-19

**Authors:** Sandra Luna-Fineman, Guillermo Chantada, Amanda Alejos, Geraldina Amador, Margarita Barnoya, Mauricio E. Castellanos, Ligia Fu, Soad Fuentes-Alabi, Verónica Girón, Marco Antonio Goenz, Carlos Maldonado, Gustavo Méndez, Rosa Amelia Morales, Roberta Ortiz, Gissela Sanchez, Matthew Wilson, Carlos Rodríguez-Galindo

**Affiliations:** ^1^ Children’s Hospital Colorado, University of Colorado, Denver, CO; ^2^ Unidad Nacional de Oncología Pediátrica, Guatemala City, Guatemala; ^3^ Hospital J.P. Garrahan, Buenos Aires, Argentina; ^4^ Hospital Universitario, Tegucigalpa, Honduras; ^5^ Hospital Herrera Llerandi, Guatemala City, Guatemala; ^6^ Hospital Infantil Bloom, San Salvador, El Salvador; ^7^ Hospital Infantil Manuel de Jesús Rivera–La Mascota, Managua, Nicaragua; ^8^ Centro Nacional de Oftalmología, Managua, Nicaragua; ^9^ Hospital Solca, Quito, Ecuador; ^10^ University of Tennessee Health Sciences Center, Memphis, TN; ^11^ St Jude Children’s Research Hospital, Memphis, TN

## Abstract

**PURPOSE:**

Treatment abandonment because of enucleation refusal is a limitation of improving outcomes for children with retinoblastoma in countries with limited resources. Furthermore, many children present with buphthalmos and a high risk of globe rupture during enucleation. To address these unique circumstances, the AHOPCA II protocol introduced neoadjuvant chemotherapy with delayed enucleation.

**PATIENTS AND METHODS:**

Patients with advanced unilateral intraocular disease (International Retinoblastoma Staging System [IRSS] stage I) were considered for upfront enucleation. Those with diffuse invasion of the choroid, postlaminar optic nerve, and/or anterior chamber invasion received six cycles of adjuvant chemotherapy (vincristine, carboplatin, and etoposide). Patients with buphthalmos and those with a perceived risk for enucleation refusal and/or abandonment were given two to three cycles of chemotherapy before scheduled enucleation followed by adjuvant chemotherapy to complete six cycles, regardless of pathology.

**RESULTS:**

A total of 161 patients had unilateral IRSS stage I disease; 102 underwent upfront enucleation, and 59 had delayed enucleation. The estimated 5-year abandonment-sensitive event-free and overall survival rates for the group were 0.81 ± 0.03 and 0.86 ± 0.03, respectively. The 5-year estimated abandonment-sensitive event-free survival rates for patients undergoing upfront and delayed enucleation were 0.89 ± 0.03 and 0.68 ± 0.06, respectively (*P* = .001). Compared with AHOPCA I, abandonment for patients with IRSS stage I retinoblastoma decreased from 16% to 4%.

**CONCLUSION:**

AHOPCA describes the results of advanced intraocular retinoblastoma treated with neoadjuvant chemotherapy. In eyes with buphthalmos and patients with risk of abandonment, neoadjuvant chemotherapy can be effective when followed by enucleation and adjuvant chemotherapy. Our study suggests that this approach can save patients with buphthalmos from ocular rupture and might reduce refusal of enucleation and abandonment.

## INTRODUCTION

Retinoblastoma is the most common eye tumor of childhood and represents 3% of childhood cancers.^1^ In high-income countries (HICs), the majority of children are cured by enucleation only or with a combination of chemotherapy and focal treatments for ocular preservation; most present with limited intraocular disease, and orbital and metastatic disease are rare occurrences. In this scenario, children with advanced unilateral intraocular disease can be cured with enucleation followed by pathology risk–adapted adjuvant chemotherapy.^2-6^

In contrast, in low- and middle-income countries (LMICs), retinoblastoma presents with advanced disease secondary to delayed diagnosis.^7-9^ Ocular salvage rarely is possible, and many patients have incurable metastatic disease.^10-12^ Although upfront enucleation is curative for children with intraocular disease, the presence of buphthalmos (an abnormal enlargement of the eye, usually in infants and young children and associated with advanced intraocular retinoblastoma) and refusal to accept enucleation are major limitations to cure.^13^ Buphthalmos is associated with an increased risk of microscopic tumor in the cut end of the optic nerve (and often in the sclera), and upfront enucleation also carries a risk of rupture, causing contamination of the orbit and increasing the risk of relapse.^8,13-17^ Finally, a considerable proportion of patients refuse enucleation and abandon treatment.^18^ Abandonment of therapy is defined as the failure to return to continue treatment (chemotherapy or radiation) for 4 weeks or more.^19^ Furthermore, most patients die if they do not undergo enucleation.^11,13^ This challenge requires cultural and medicolegal approaches that are seldom available in LMICs and critically affect survival.^7,13^

The Central American Association of Pediatric Oncology (AHOPCA) reported the results of its first retinoblastoma study AHOPCA I, where very advanced disease presentation was documented with a 5-year overall survival (OS) rate of 0.48 ± 0.04.^11^ In this cohort, 45 of 171 patients had unilateral stage I disease, and 16% abandoned therapy. Over subsequent years, the participating institutions embarked on a regionwide initiative (AHOPCA II) aimed at enhancing diagnosis and establishing a risk-adapted therapy that involved group discussion of all patients. Herein, we present the results of a risk-adapted approach for patients with advanced intraocular unilateral retinoblastoma. The standard approach of upfront enucleation and adjuvant therapy adjusted to pathology risk factors was modified for patients who presented with buphthalmos and for those who refused upfront enucleation or were at risk of abandonment to include neoadjuvant chemotherapy and delayed enucleation.

## PATIENTS AND METHODS

From April 2007 to December 2015, 473 patients were enrolled in the AHOPCA II retinoblastoma protocol. Fifty of these 473 patients were subsequently considered ineligible for this study. Four patients had been previously treated, and 46 had insufficient institutional review board approval. In particular, the Costa Rica investigators removed their patients from the study (n = 46) because of a technical misunderstanding with their institutional review board; the study was registered in Costa Rica as a single-center study instead of as a multicenter study; therefore, the investigators were not authorized to include their patients in this study. Hence, the remaining 423 eligible patients participated in the AHOPCA II retinoblastoma protocol (Guatemala, 218 patients; Honduras, 83 patients; El Salvador, 53 patients; Nicaragua, 69 patients). At diagnosis, patients had an examination under anesthesia by a pediatric ophthalmologist. All patients were assessed for eligibility and staged at diagnosis for intraocular disease (Reese-Ellsworth and International Intraocular Retinoblastoma Classification) and metastatic disease (St Jude and International Retinoblastoma Staging System [IRSS]).^20^ Imaging included computed tomography scan or magnetic resonance imaging of the orbits and brain. A lumbar puncture and bone marrow biopsy were performed to exclude metastatic disease. All patients were assessed for risk of abandonment of therapy using local and institutional standard practices.^21,22^ The determinants of treatment abandonment for retinoblastoma in Central America are mutilating surgery,^23^ low education, low socioeconomic status,^19,24^ and long travel time to the cancer center.^25^ The interventions varied among institutions, but in general, they included psychosocial evaluation and counseling, meeting other families affected by retinoblastoma who completed treatment, help with expenses for transportation, lodging near the hospital, and a basic food basket for the family provided by the nongovernmental organizations associated with the cancer centers. The study complied with the principles of the Declaration of Helsinki and was approved by the institutional research committee of each participating institution. Parents or legal guardians signed the informed consent required for enrollment and therapy.

**FIG A1.**
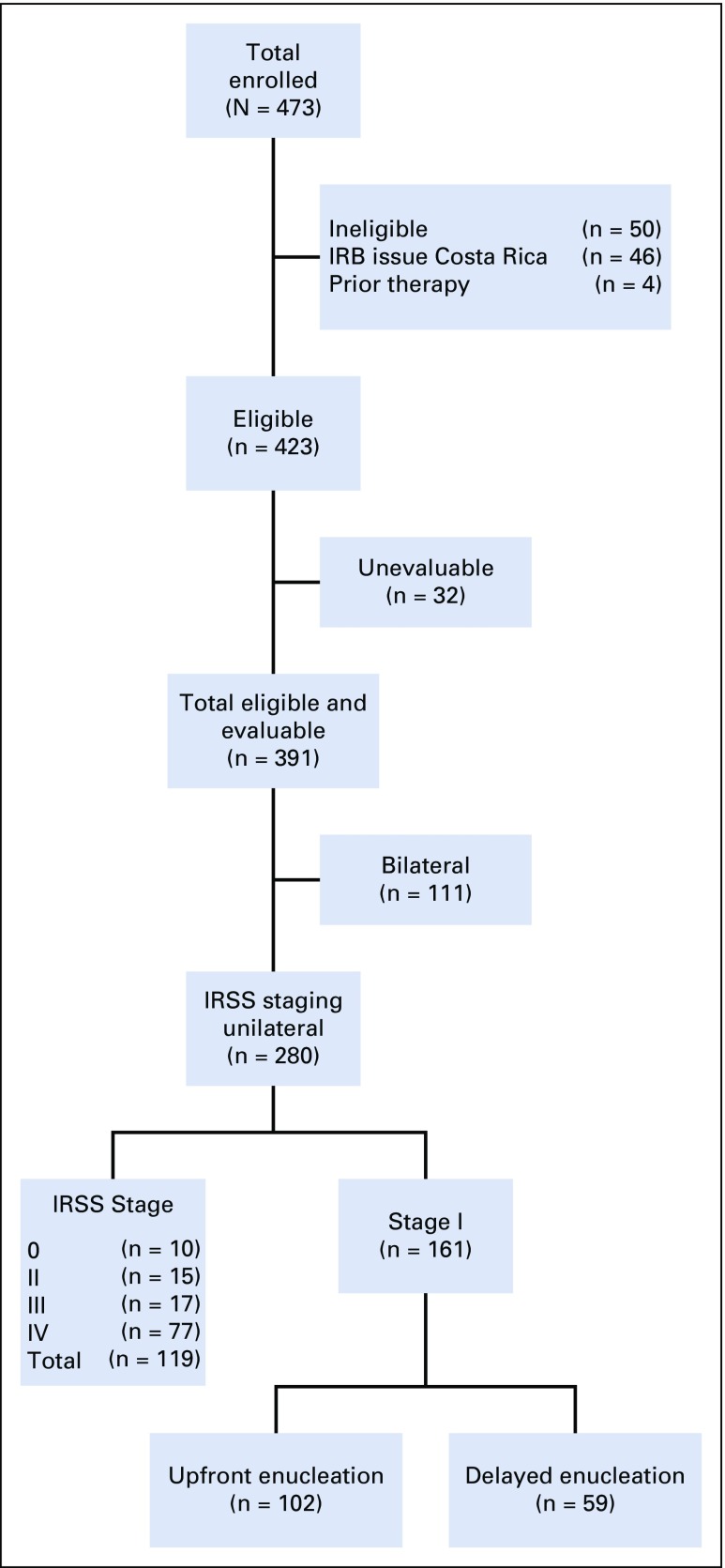
Patient flow diagram. IRB, institutional review board; IRSS, International Retinoblastoma Staging System.

Of the 423 patients (Appendix Fig A1, online only), 32 were not evaluable: 27 (84%) refused all treatments upfront (enucleation, chemotherapy, or observation) and were lost to follow-up, three patients with metastasis had palliation, one transferred to another hospital, and one had incomplete data. Of the 391 remaining eligible, evaluable patients, 111 had bilateral disease, and 280 had unilateral disease. Among the patients with unilateral disease, 10 (4%) were considered candidates for ocular preservation (stage 0), 161 (61%) had intraocular disease by imaging and were considered candidates for upfront enucleation (IRSS stage I for the purposes of this study), 15 (5%) had stage II disease, 17 (5%) had stage III disease, and 77 (25%) had stage IV disease (Appendix Fig A1).

This report focuses on the 161 patients with newly diagnosed, untreated unilateral intraocular retinoblastoma assigned clinically as IRSS stage I. An evaluation of the feasibility of upfront enucleation was performed. Patients with buphthalmos (defined as an eye globe increased in size with no evidence of extraocular dissemination by imaging studies) and those judged to be at risk for refusal or abandonment of therapy received neoadjuvant chemotherapy with delayed enucleation. This allowed for the implementation of support and counseling as mentioned earlier. All other patients underwent upfront enucleation.

After upfront enucleation, patients were classified according to pathology risk factors to tailor adjuvant therapy. Low-risk pathology was defined as the absence of a massive invasion of the choroid, invasion of the anterior chamber, optic nerve beyond the lamina cribrosa, or sclera. Intermediate-risk pathology was defined as the presence of a massive invasion of the choroid or anterior chamber, postlaminar invasion of the optic nerve without tumor at the cut end, or intrascleral involvement. The histopathology was interpreted by the local pathologist without central pathology review.

Patients undergoing upfront enucleation with low-risk pathology had no other intervention and were followed with examination under anesthesia and imaging studies every 4 to 6 months as clinically indicated. Those with intermediate-risk pathology received six cycles of adjuvant chemotherapy with vincristine 1.5 mg/m^2^ and carboplatin 500 mg/m^2^ on day 1 and etoposide 100 mg/m^2^ on days 1 to 3 (doses were adjusted per kilogram body weight if less than 10 kg) every 4 weeks.

Patients with buphthalmos or perceived to be at risk for abandonment of therapy were given two to three cycles of neoadjuvant chemotherapy as described for patients with intermediate-risk pathology before scheduled enucleation. Thereafter, adjuvant chemotherapy was administered to complete a total of six cycles, irrespective of pathology. Radiation therapy was added for patients with high-risk pathology, defined as extrascleral involvement or disease at the cut end of the optic nerve (50 Gy to the orbit and extended to the chiasm if the optic nerve was involved). Online meetings through Cure4Kids^26^ were regularly scheduled throughout the development and implementation of the protocol. All patients were discussed for consensus staging and treatment. Clinical data were collected on POND4Kids,^27^ an online pediatric oncology database with standardized forms specific for the study. All the events were prospectively recorded and reviewed yearly.

### Statistical Methods

A primary objective of AHOPCA II was to obtain descriptive data about the feasibility of using chemotherapy before enucleation in patients with buphthalmos and risk of abandonment of therapy. This report presents the results for this objective. The primary end point for the study was abandonment-sensitive event-free survival (EFS) and OS. EFS was defined as the time from diagnosis to first event (relapse, progression, abandonment, or death) or last follow-up. Relapse was defined as the return of disease after the patient had been declared disease free at completion of therapy. Progressive disease was defined as tumor advancement during therapy. Abandonment of therapy was defined as the failure to return to continue treatment of 4 weeks or more.^19^ Abandonment-sensitive EFS estimates were calculated using the Kaplan-Meier method.^28^ OS was defined as the time from diagnosis to death or last follow-up for those who were alive. Log-rank test was used to compare survival curves. OS was compared with the previous study (AHOPCA I, 1999 to 2004).^11^ Survival rates are presented as proportions ± SE of the estimate.

## RESULTS

### Demographics and Staging

The characteristics of the 161 patients with IRSS stage I disease are listed in Table 1. Twenty-eight percent (45 of 161) were older than 3 years of age. Most (142 of 191; 88%) self-identified as Latino/mestizo, and 17 (11%) identified as indigenous or Amerindian (all from Guatemala). One hundred two patients (64%) underwent upfront enucleation, and 59 (36%) had delayed enucleation with neoadjuvant chemotherapy. Of the latter, 21 had buphthalmos, and 38 received chemotherapy because of a risk of abandonment of therapy. Of 161 patients, 142 (88%) had advanced intraocular disease by the International Intraocular Retinoblastoma Classification system (group C, D, and E disease), and 153 (95%) by the Reese-Ellsworth system (groups 4 and 5; data not shown).

**TABLE 1.**
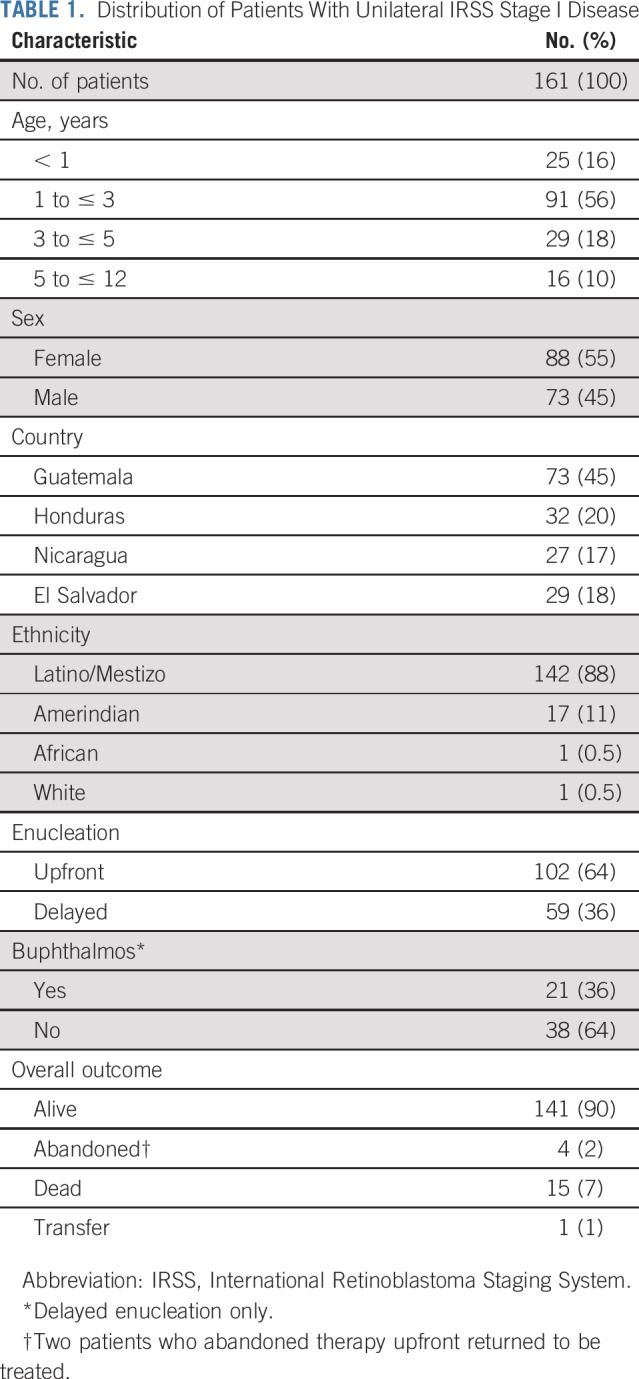
Distribution of Patients With Unilateral IRSS Stage I Disease

### Pathology

**FIG 1.**
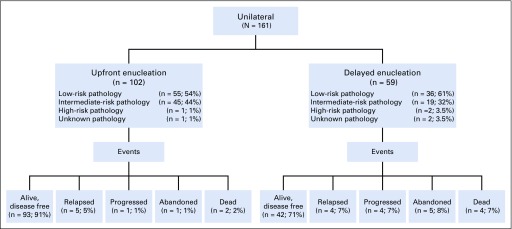
Patient distribution.

None of the eyes ruptured during enucleation. Upfront enucleation was performed on 102 patients, and 59 patients had delayed enucleation (Fig 1). Of the 102 patients who underwent upfront enucleation, 55 (54%) had low-risk pathology and did not require chemotherapy, and 45 (44%) had intermediate-risk pathology that required adjuvant chemotherapy. One patient had high-risk pathology, and one had no pathology available and received chemotherapy. Of the 59 patients who received preoperative chemotherapy and delayed enucleation, only two (3.5%) had high-risk pathology, and in one patient, pathology was unknown. Both patients with high-risk pathology abandoned therapy.

### Abandonment of Therapy

Six patients (4%) abandoned therapy during treatment despite psychosocial interventions or chemotherapy before enucleation. Two of them subsequently returned, with both belonging to the delayed enucleation group (one because of a risk of abandonment and one with buphthalmos). Finally, four (2%) of 161 patients never returned. Of these, one is alive at 7 years of follow-up and the second was lost to follow-up at 1 year from diagnosis. All abandonments occurred during the first 6 months of therapy (one from the upfront enucleation group and five from the delayed enucleation group). One more patient abandoned therapy after relapse and did not return.

### Toxicity

Overall, the chemotherapy was well tolerated, with the expected myelosuppression. However, there were six deaths (4%) during the study: four patients had a documented death as a result of toxicity (one as a result of hemorrhage, three as a result of infection), and two patients died at home with no additional information available.

### Events

A combined total of 26 (16%) of 161 patients had an event (Fig 1). As discussed previously, six patients (4%) abandoned therapy and six (4%) died as a first event. Fourteen patients experienced relapse or progression (six [6%] in the upfront enucleation group and eight [14%] in the delayed enucleation group). Nine (6%) had an extraocular relapse (five to the orbit, three to CNS, and one to an unknown site), and five (3%) progressed while on therapy (two to the orbit, one to the brain, and two to unknown sites). Five of these 14 patients are still alive. Eight patients were lost to follow-up at a median time of 1.1 years (0.4 to 1.8 years).

### Outcomes

**FIG 2.**
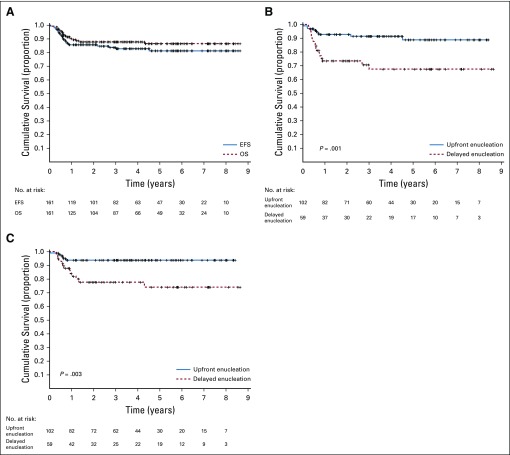
(A) Five-year abandonment-sensitive event-free survival (EFS) and overall survival (OS) for the entire group. Five-year (B) abandonment-sensitive EFS and (C) OS for patients who underwent upfront enucleation and delayed enucleation.

The 5-year abandonment-sensitive EFS and OS estimates for the entire group were 0.81 ± 0.03 and 0.86 ± 0.03, respectively (Fig 2A). As shown in Figure 2B, the 5-year abandonment-sensitive EFS for the patients who had upfront enucleation and for those who underwent delayed enucleation were 0.89 ± 0.04 and 0.68 ± 0.07, respectively (*P* = .003). The 5-year abandonment-sensitive OS estimates for the upfront enucleation and the delayed enucleation groups were 0.94 ± 0.02 and 0.74 ± 0.0, respectively (*P* < .001; Fig 2C).

**FIG 3.**
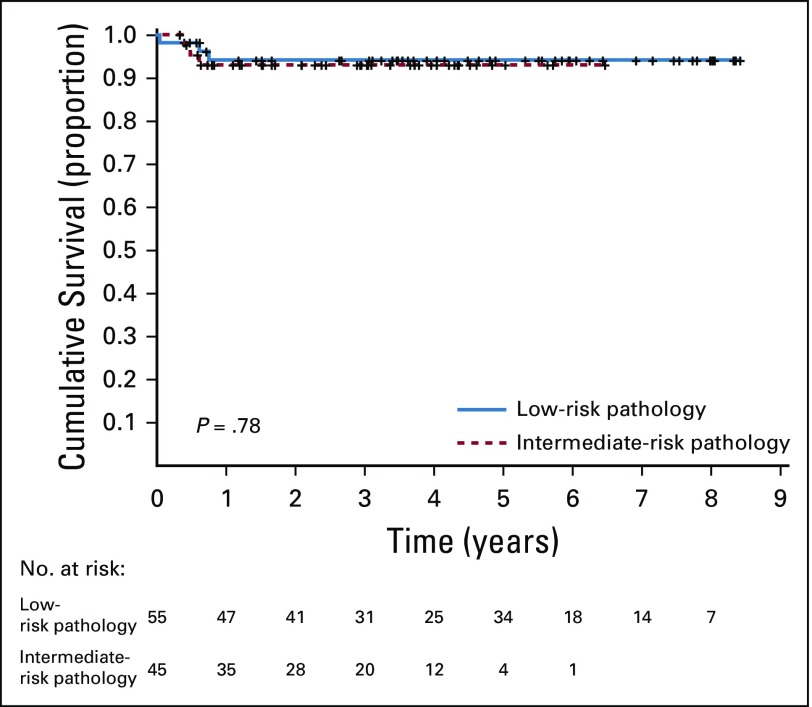
Five-year abandonment-sensitive overall survival estimates for patients with low-risk and intermediate-risk pathology for the upfront enucleation group.

**FIG 4.**
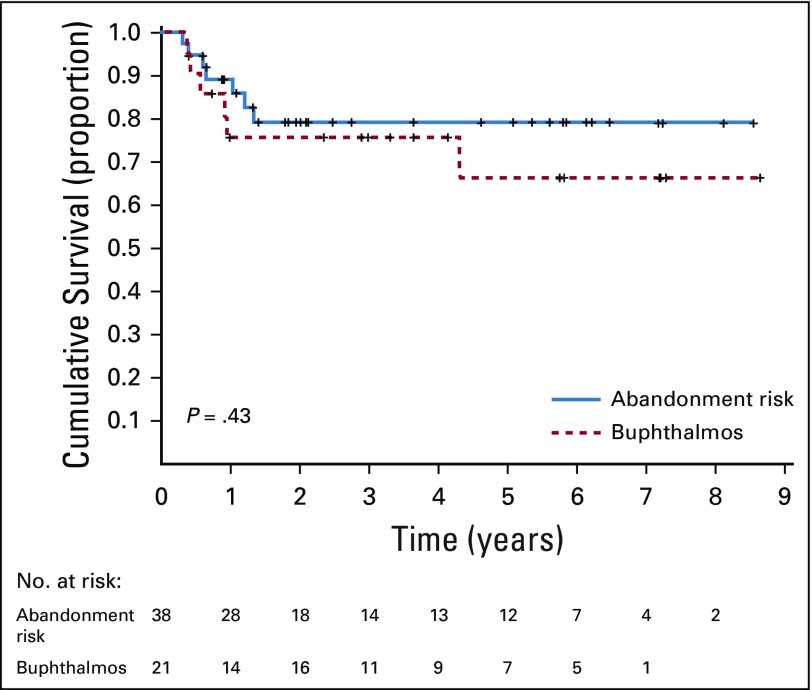
Five-year abandonment-sensitive overall survival estimates for patients with buphthalmos and risk of abandonment for the delayed enucleation group.

For the upfront enucleation group, the 5-year abandonment-sensitive EFS estimates for patients with low-risk pathology and intermediate-risk pathology were 0.90 ± 0.04 and 0.77 ± 0.14, respectively (*P* = .72; data not shown), and the OS estimates were 0.94 ± 0.03 and 0.93 ± 0.04, respectively (*P* = .78; Fig 3). Among the 55 patients with low-risk pathology, there were five (9%) events (one death as a result of complications of enucleation and four relapses [three in the orbit and one in the CNS (two patients with orbital relapse were rescued and are alive)]). Among the 45 patients with intermediate-risk pathology after upfront enucleation, there were four (9%) events (one patient died as a result of infection, one abandoned therapy, and two experienced a relapse in the orbit; one patient is alive). For the delayed enucleation group, the 5-year abandonment-sensitive EFS estimates for patients with buphthalmos and risk of abandonment of therapy were 0.54 ± 0.12 and 0.77 ± 0.07 (*P* = .14), respectively (data not shown), and the OS estimates were 0.66 ± 0.12 and 0.80 ± 0.07, respectively (*P* = .43; Fig 4).

For the 21 patients who received a delayed enucleation because of buphthalmos, 10 had low-risk pathology, 10 had intermediate-risk pathology, and one had high-risk pathology. Nine (43%) of the 21 patients with buphthalmos had an event; four (19%) experienced relapse (two in the orbit and two in the CNS), three (14%) abandoned therapy, and two (10%) died as a result of toxicity (one as a result of sepsis and one as a result of CNS hemorrhage).

For the 38 patients who received delayed enucleation because of risk of abandonment, 27 had low-risk pathology, nine had intermediate-risk pathology, and one had high-risk pathology; pathology for one patient was not available. Eight (21%) of these 38 patients had an event: four (10%) had an extraocular relapse (two in the orbit, one in the CNS, and one unknown) and died, two (5%) abandoned therapy, and two (5%) died as a result of an unknown cause.

## DISCUSSION

We report the results of the second protocol conducted by the AHOPCA group for children with unilateral intraocular retinoblastoma (IRSS stage I). The 5-year abandonment-sensitive EFS and OS for the entire group were 0.81 ± 0.03 and 0.86 ± 0.03, respectively. In recognition of the limitations of cross-study comparisons, this seems to represent an improvement from our previous AHOPCA I study of a 5-year OS of 0.70 ± 0.06.^11^

As shown here, the standards of care developed by retinoblastoma centers and consortia in HICs may not always be applicable to LMICs; the advanced presentation, socioeconomic barriers, and limited access to care that define retinoblastoma in these countries call for resource-adapted treatments. However, those adapted approaches should be evaluated properly in the context of a clinical trial to measure their impact and usefulness. In this study, we addressed two circumstances unique to resource-limited countries: the management of patients who present with buphthalmos and the approach for patients at risk for abandonment of therapy and for whom preoperative chemotherapy would allow for more time for counseling while keeping the disease under control. To our knowledge, this LMIC multinational study is the first to describe prospectively the use of delayed enucleation with neoadjuvant chemotherapy for the management of advanced unilateral intraocular retinoblastoma in resource-limited settings.

Using this approach, abandonment of therapy was reduced from 16% in the previous protocol^11^ to 4% in the current study, and those classified as having a high risk of abandonment had a 5-year OS of 0.80 ± 0.07. Although it is not possible to compare this group of patients with a similar cohort in the prior study because of the lack of a dedicated approach, the favorable outcomes in AHOPCA II probably reflect the multidisciplinary interventions implemented. As suggested by Zhao et al,^29^ the use of delayed enucleation with neoadjuvant chemotherapy does not allow for risk assignment on the basis of pathology risk factors. Possibly, our patients considered at high risk of abandonment of therapy before enucleation had more-advanced intraocular disease and higher-risk pathology, conceivably as a result of the same socioeconomic factors that cause delays in diagnosis. Although outcomes for this group of patients were inferior to those who underwent upfront enucleation, we still consider that this strategy was effective for this population given that most patients who refuse enucleation ultimately die as a result of retinoblastoma.^7,13,23^ In our previous study,^11^ 41% of patients who abandoned therapy died.

The cohort that received delayed enucleation also included children who presented with buphthalmos for whom a risk of rupture was considered high. Chantada et al^15^ described 20% of these patients treated with upfront enucleation to have tumor at the margin of the optic nerve, and the use of preoperative chemotherapy has been proposed by others.^14,30^ The outcome for this group was inferior, with a 5-year OS of 0.70 ± 0.11, which probably reflects the high tumor burden associated with buphthalmos. In AHOPCA I,^11^ only 45 patients were reported to have unilateral, stage I disease. Of these, 22 (49%) had upfront enucleation, and their OS was 0.82 ± 0.09. Twenty-three (51%) had delayed enucleation, and their OS was 0.60 ± 0.13. Although we could not perform accurate comparisons with the prior study because of the lack of complete documentation of buphthalmos, these results are comparable to the Pérez et al^31^ Grupo de America Latina de Oncología Pediátrica cohort with buphthalmos treated with neoadjuvant chemotherapy, where one of seven patients with buphthalmos experienced a relapse.

The outcomes for children who underwent upfront enucleation were highly satisfactory; OS for patients with stage I disease increased from 0.70 ± 0.06 in AHOPCA I to 0.94 ± 0.02 in AHOPCA II. Survival for patients with low- and intermediate-risk pathology was excellent, with a 5-year OS of 0.94 ± 0.03 and 0.93 ± 0.04, respectively, comparable with results from HICs.^32-34^ Only 6% of patients who underwent upfront enucleation had extraocular relapse or progression; this may be improved further with central pathology review for better risk-adapted treatment. In retinoblastoma, histopathology interpretation is of the upmost importance^34,35^; specific guidelines for the definition of the involvement of the coats and optic nerve were part of the protocol, but experience of the local pathologists still lags. A probability exists that the pathology reports of the centers were not reliable and that adjudication of risk was inappropriate. Additional training of pathologists and central review to improve quality are necessary.

Six deaths as a result of toxicity (3%) occurred in our study population, all during the first 3 years of the study. This finding is not uncommon in LMICs^36^ because distance from the centers, local hospitals with the knowledge of immediate treatment of sepsis, and poor networks of care affect outcome.^37^ More-intensive chemotherapy regimens that have shown efficacy in preventing extraocular relapse may not be applicable in resource-limited settings.^15,32^ An effort to address the ability of resource-constrained centers through the design of regimens appropriate to the country has been addressed by the International Society of Pediatric Oncology.^38,39^ Hence, the use of adjuvant therapy in our setting should be determined carefully.

We must underline the importance of monitoring new standards of care in LMICs. The implementation of systems for data quality assessment and prospective evaluation of care as well as individualized case discussions are critical for the development of evidence-based treatments. Regular Web-based conferences, case discussions, and data monitoring were conducted throughout the duration of the study. Using this approach, standardization of therapy with steep learning curves can be addressed easily in a forum of collaboration and mutual support.

In conclusion, we were able to implement an effective risk-based and resource-adapted treatment of intraocular unilateral retinoblastoma in a multinational setting and to show that delayed enucleation after neoadjuvant chemotherapy is feasible in the management of advanced intraocular retinoblastoma with buphthalmos using a multidisciplinary approach to prevent abandonment of therapy. Measures to improve early diagnosis of retinoblastoma in Central America are being implemented, and a prospective clinical trial with central pathology review to refine risk-adapted regimens is planned.
